# An improved model using convolutional sliding window-attention network for motor imagery EEG classification

**DOI:** 10.3389/fnins.2023.1204385

**Published:** 2023-08-15

**Authors:** Yuxuan Huang, Jianxu Zheng, Binxing Xu, Xuhang Li, Yu Liu, Zijian Wang, Hua Feng, Shiqi Cao

**Affiliations:** ^1^School of Computer Science and Technology, Donghua University, Shanghai, China; ^2^Department of Neurosurgery and State Key Laboratory of Trauma, Burn and Combined Injury, Southwest Hospital, Third Military Medical University (Army Medical University), Chongqing, China; ^3^Department of Orthopaedics of TCM Clinical Unit, The Sixth Medical Center, Chinese PLA General Hospital, Beijing, China

**Keywords:** EEG, motor imagery, brain computer interface, deep learning, CNN, attention

## Abstract

**Introduction:**

The classification model of motor imagery-based electroencephalogram (MI-EEG) is a new human-computer interface pattern and a new neural rehabilitation assessment method for diseases such as Parkinson's and stroke. However, existing MI-EEG models often suffer from insufficient richness of spatiotemporal feature extraction, learning ability, and dynamic selection ability.

**Methods:**

To solve these problems, this work proposed a convolutional sliding window-attention network (CSANet) model composed of novel spatiotemporal convolution, sliding window, and two-stage attention blocks.

**Results:**

The model outperformed existing state-of-the-art (SOTA) models in within- and between-individual classification tasks on commonly used MI-EEG datasets BCI-2a and Physionet MI-EEG, with classification accuracies improved by 4.22 and 2.02%, respectively.

**Discussion:**

The experimental results also demonstrated that the proposed type token, sliding window, and local and global multi-head self-attention mechanisms can significantly improve the model's ability to construct, learn, and adaptively select multi-scale spatiotemporal features in MI-EEG signals, and accurately identify electroencephalogram signals in the unilateral motor area. This work provided a novel and accurate classification model for MI-EEG brain-computer interface tasks and proposed a feasible neural rehabilitation assessment scheme based on the model, which could promote the further development and application of MI-EEG methods in neural rehabilitation.

## 1. Introduction

The electroencephalogram (EEG) is a non-invasive diagnostic technique that records brain activity by placing electrodes on the scalp to detect electrical signals from neurons in the brain. It can be used to diagnose various neurological disorders, such as epilepsy, sleep disorders, and cerebrovascular accidents. Additionally, it can decode and analyze the intentions of the brain for controlling external mechanical devices. The brain-computer interface (BCI) represents a new generation of human-computer interaction that harnesses the power of EEG devices to capture human neural signals, which are then analyzed and classified using pattern recognition algorithms to control computers (Vaid et al., 2015). Motor imagery is a type of EEG signal that arises from the mental simulation or imagination of movements, resulting in neural patterns like those observed during actual physical movement. This EEG signal can be utilized for brain-machine communication, such as controlling prosthetics or wheelchairs, and also holds potential applications in neurorehabilitation (Abiri et al., 2019).

MI is now widely used in various neurorehabilitation training programs (Jeunet et al., 2015; Ron-Angevin et al., 2017; Paris-Alemany et al., 2019). In the training program, motor imagery promotes the regeneration of brain neurons and connectivity by internally simulating the movements of specific muscles. MI could also improve the coordination patterns during the formation process of motor skills and provide muscles with additional opportunities for skill practice, which aids in learning or regaining control of actual movements. It has been utilized to improve the muscle control and recovery abilities of patients with Parkinson's disease, post-stroke sequelae, post-brain injury sequelae, and joint diseases (Williams et al., 2004; Moseley, 2006; Tamir et al., 2007; Zimmermann-Schlatter et al., 2008). The classification of MI signals recorded by EEG (MI-EEG) was also used in the neurorehabilitation assessment in the training programs, which was limited by the classification performance of the MI-EEG algorithms (Chen et al., 2022; Cuomo et al., 2022; Binks et al., 2023).

The traditional classification frameworks have utilized feature extraction techniques to manually extract features in the time-frequency domain of MI-EEG signals and subsequently classified the signals using machine learning algorithms, such as Filter Bank Common Spatial Pattern (FBCSP) (Chin et al., 2009), Fast Fourier Transform (FFT) (Wang et al., 2018b), Wavelet Transform (Qin and He, 2005), Support Vector Machines (SVM) (Selim et al., 2018), Linear Discriminant Analysis (LDA) (Steyrl et al., 2014), and K-Nearest Neighbor (KNN) (Bhattacharyya et al., 2010). However, these methods have high requirements for manual feature design and are greatly affected by designers and data, which is not conducive to the application and promotion of various scenarios, including neurorehabilitation assessment.

In recent years, deep learning algorithms, which have excelled in the fields of vision and language research, have significantly improved the classification performance of MI-EEG classification. Using deep learning algorithms, classifiers could automatically extract features without manual feature extraction or reliance on specific MI-EEG data. Deep learning methods such as Multi-Layer Perceptron (MLP) (Chatterjee and Bandyopadhyay, 2016; Samuel et al., 2017), Convolutional Neural Networks (CNN) (Dai et al., 2019; Hou et al., 2020; Li et al., 2020; Zancanaro et al., 2021; Altuwaijri et al., 2022), Deep Belief Networks (DBN) (Xu and Plataniotis, 2016), Recurrent Neural Networks (RNN) (Luo et al., 2018; Kumar et al., 2021), as well as Long Short-Term Memory (LSTM) in combination with CNN or RNN for spatiotemporal features (Wang et al., 2018a; Khademi et al., 2022), have been successfully proposed for MI-EEG tasks. The classification performance of these methods could far outperform traditional machine learning methods.

Nowadays, attention mechanisms with dynamic spatio-temporal feature extraction for deep learning are demonstrated to have strong adaptive feature extraction capabilities, which have been shown to help improve performance in various machine learning tasks (Bahdanau et al., 2014). Within attention mechanisms, the multi-head self-attention model has dominated the development of the most advanced artificial intelligence algorithms (Vaswani et al., 2017). Currently, a few attention-based deep learning algorithms have been proposed for EEG signal processing, and have been found to have breakthrough performance in epilepsy detection, emotion recognition, MI classification, and other tasks (Zhang et al., 2020; Amin et al., 2022). For example, Xie et al. (2022) have proposed a novel approach that utilizes multi-head self-attention combined with position embedding to enhance the classification performance of EEG on the Physionet dataset, achieving an accuracy of 68.54%. Furthermore, Altuwaijri and Muhammad (2022) have employed channel attention and spatial attention mechanisms to capture temporal and spatial features from EEG signals on the BCI-2a dataset, resulting in an accuracy of 83.63%. However, these methods lack comprehensive integration of multi-scale spatiotemporal features and also neglect adaptive attention selection for global features (Al-Saegh et al., 2021; Altaheri et al., 2021). Both defects may reduce the feature learning and selection abilities of the MI-EEG model and affect its performance.

To solve these problems, this article proposes a model for MI-EEG classification called the convolutional sliding window-attention network (CSANet). The model consists of three components. First, a convolution block consisting of multi-layered convolutional, pooling, and normalization layers for extracting spatiotemporal features was proposed to extract the spatiotemporal features of the EEG signal preliminarily. Second, a sliding window block with continuous and dilated sliding windows was proposed to further combine the feature tokens with local and global context information and the token of window type. Finally, an attention block with local and global attention mechanisms was proposed to highlight effective features, which was followed by a classifier consisting of fully connected layers. The CASNet was evaluated in two commonly used MI-EEG datasets and was demonstrated to outperform the state-of-the-art (SOTA) models. The plausible application framework of the accurate CSANet model in neurorehabilitation assessment was also proposed in the discussion chapter. The main contributions to this work are listed as follows:

1. This article proposes a novel deep learning model for MI-EEG tasks that utilize multi-scale feature extraction modules with convolutional layers and sliding windows and feature optimization selection modules using attention mechanisms. The proposed model outperformed SOTA models in two commonly used MI-EEG datasets.
2. The spatiotemporal convolutional, continuous, and dilated sliding windows were proposed to extract effective correlated features from EEG signals to solve the problem of simple feature scale.
3. Local and global multi-head self-attention mechanisms were utilized to enhance the adaptive feature selection ability of different scale information associations in EEG signals between individuals.
4. A plausible application framework of the CASNet model was proposed to provide a possible solution for the neurorehabilitation assessment based on the brain-computer interface.

## 2. Methods

**Figure 1:**
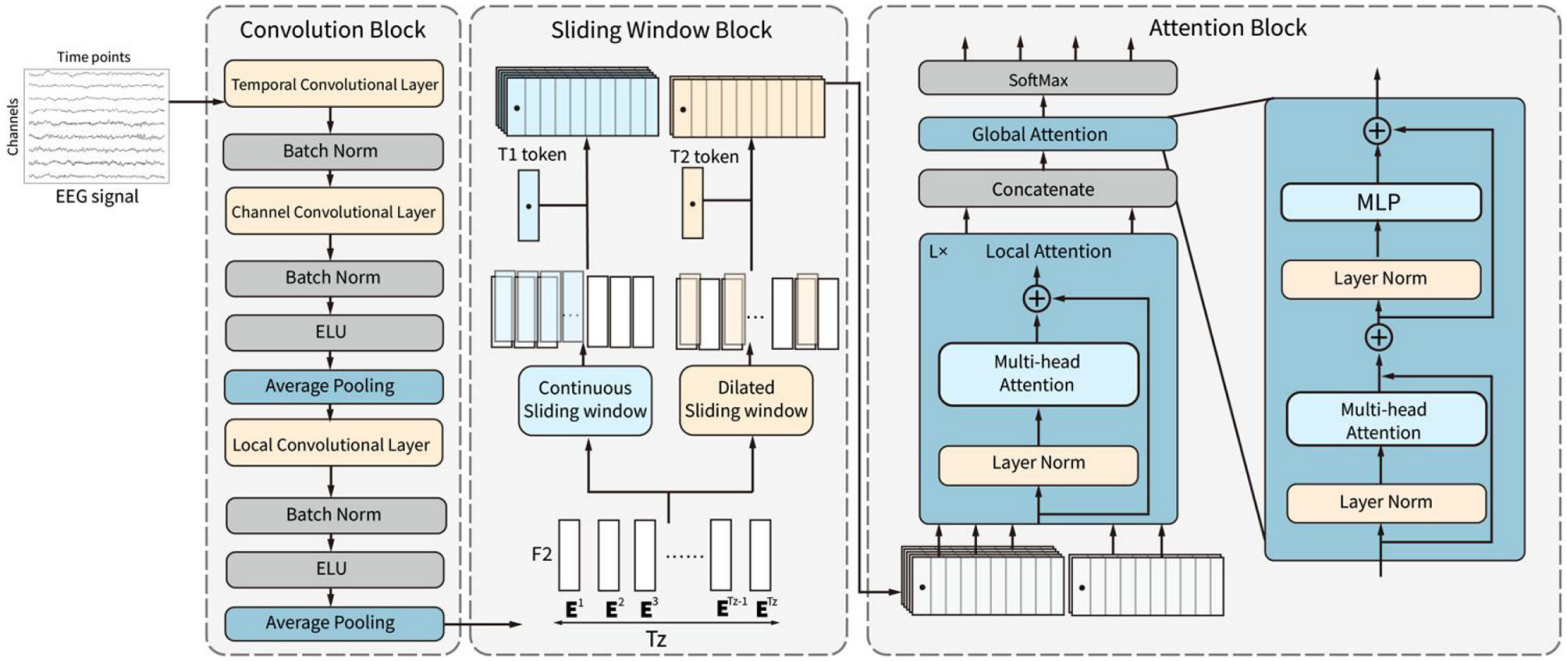
The structure of the proposed CSANet. It includes three blocks, which are the convolution block, the sliding window block, and the attention block.

The framework of the proposed CSANet model is demonstrated in Figure 1. The model comprises three sequential blocks: the convolutional block, the sliding window block, and the attention block. The convolutional block consists of three convolutional layers and two pooling layers. It extracts features from EEG signals in the time domain using convolutional layers for temporal, channel, and local feature extraction. The output feature sequence is then input into the sliding window block, which is composed of continuous and dilated sliding windows. This block extracts the local and global context information of feature sequences through two different sliding windows to improve the richness of feature expression. Finally, the features in sliding windows are adaptively selected in the attention block, in which the features in each sliding window are adaptively weighted using local attention according to the feature relationships within the window. After features in all windows are merged, global attention is utilized to weigh the features again according to the relationships between all features. The effective features are highlighted through the two-stage attention sub-blocks. Finally, two fully connected layers with SoftMax activation are used to convert the input EEG signals into the probability of each category.

### 2.1. Convolution block

**Figure 2:**
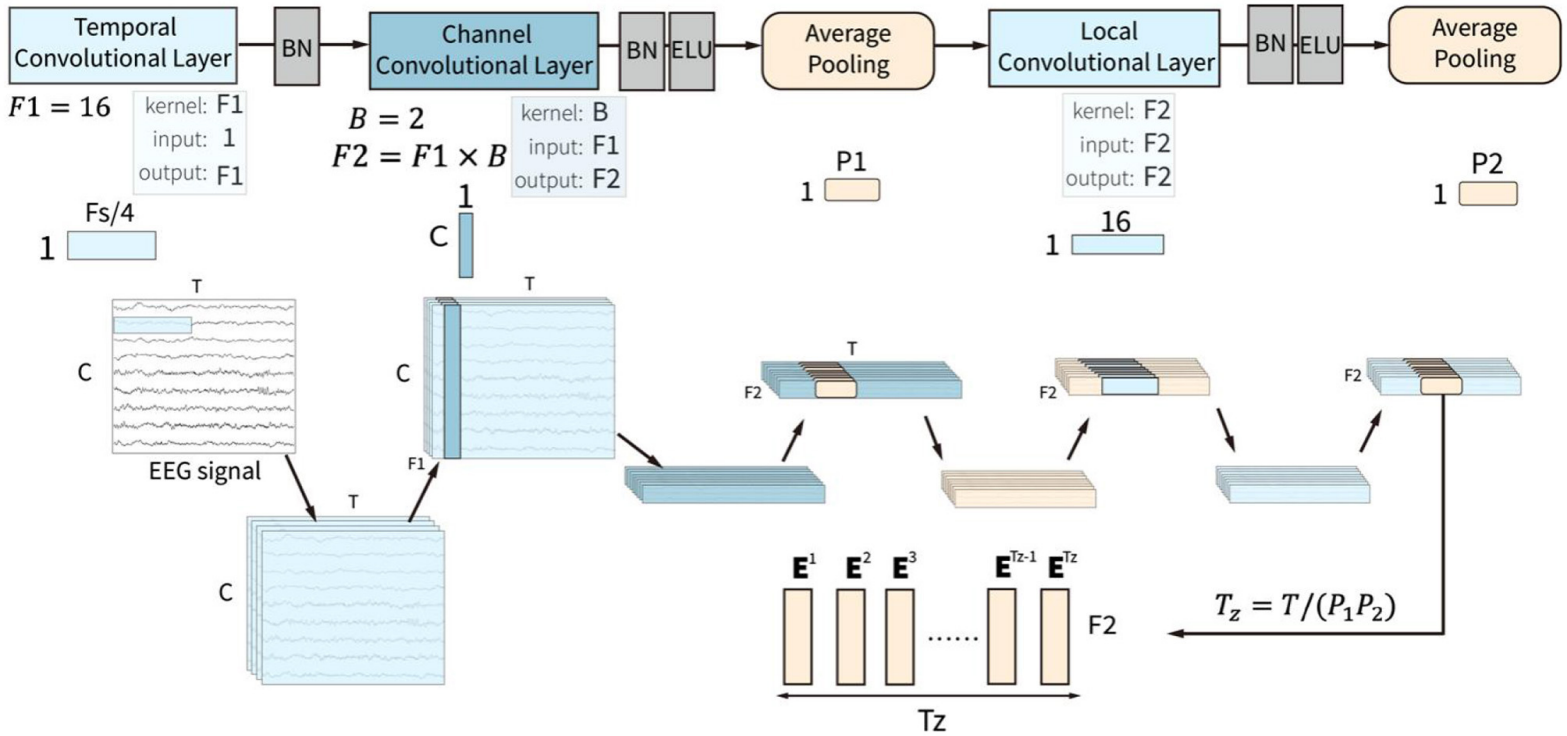
The input of the convolution block is a two-dimensional matrix with channels (C) and time points (T), which goes through three convolution layers, which are temporal, channel, and local convolutional layers, and two pooling layers.

This convolution block is similar to the feature extraction module in the ACTNet (Altaheri et al., 2022). The convolution block is composed of temporal, channel, and spatial convolutional layers and two average pooling layers, as shown in Figure 2. The three convolutional layers sequentially extract and fuse the temporal, channel, and spatial features of EEG signals to form the effective feature sequence.

The temporal convolutional layer receives the raw EEG signal with *T* time points and *C* channels in the time domain and extracts features along the time points in every single channel. It uses *F*_1_ convolutional kernel with a kernel size of 1 × *F*_*s*_/4. *F*_*s*_ is the sampling rate of the EEG signals, which means that each convolutional kernel extracts the temporal patterns within 1/4 seconds. The filters in this layer slide over the time axis and extract low-level temporal features at all the time points, which lays the foundation for the construction of high-level temporal features in the sliding window block. A batch normalization layer follows the temporal convolutional layer.

The channel convolutional slayer receives the normalized low-level temporal features. The layer utilized the depth-wise convolutional layer to extract channel-spatial features from the input features for all the temporal features from the same time points. This layer used *B* depth-wise convolutional kernels with a kernel size of *C*×1, where C represents the number of channels. Each depth-wise convolutional kernel is applied to each input feature map and outputs *B* feature maps, which means that for the input *F*_1_ feature maps, the layer outputs *F*_2_ = *F*_1_×*B* feature maps. This approach allows the channel convolutional layer to capture the valuable information of inter-channel dependencies in the EEG signals. A batch normalization layer and an Exponential Linear Unit (ELU) activation function follow the convolutional layer. Then, an average pooling layer with a pooling size of 1 × *P*_1_ is used to compress the features. This layer reduces the spatial dimensionality of the features obtained from the previous layer while retaining important information.

The local convolutional layer is designed to integrate the local spatiotemporal features with a convolutional kernel size of 1 × 16. A batch normalization, an ELU activation function, and an averaging pooling layer process the output feature sequence after the local convolutional layer. The pooling layer is set with a pooling size of 1 × *P*_2_. The size of output features is *F*_2_×*T*_*z*_, where *T*_*z*_ = *T*/(*P*_1_*P*_2_). The output features could be deemed as *T*_*z*_ sequential embedding token with *F*_2_ features. The token embedding sequence **z**_**c**_ is defined as:


(1) $$\begin{array}{l} z_{c}\;=\;[E^{1},E^{2},\cdots ,E^{T_{z}}]\\ E^{i}\in {\mathbb{R}}^{F_{2}},1\leq i\leq T_{z} \end{array}$$

**E**^*i*^ are the extracted token embeddings for *i*th token. The token embedding sequence is then input into the sliding window block to extract the high-level temporal features.

### 2.2. Sliding window block

**Figure 3:**
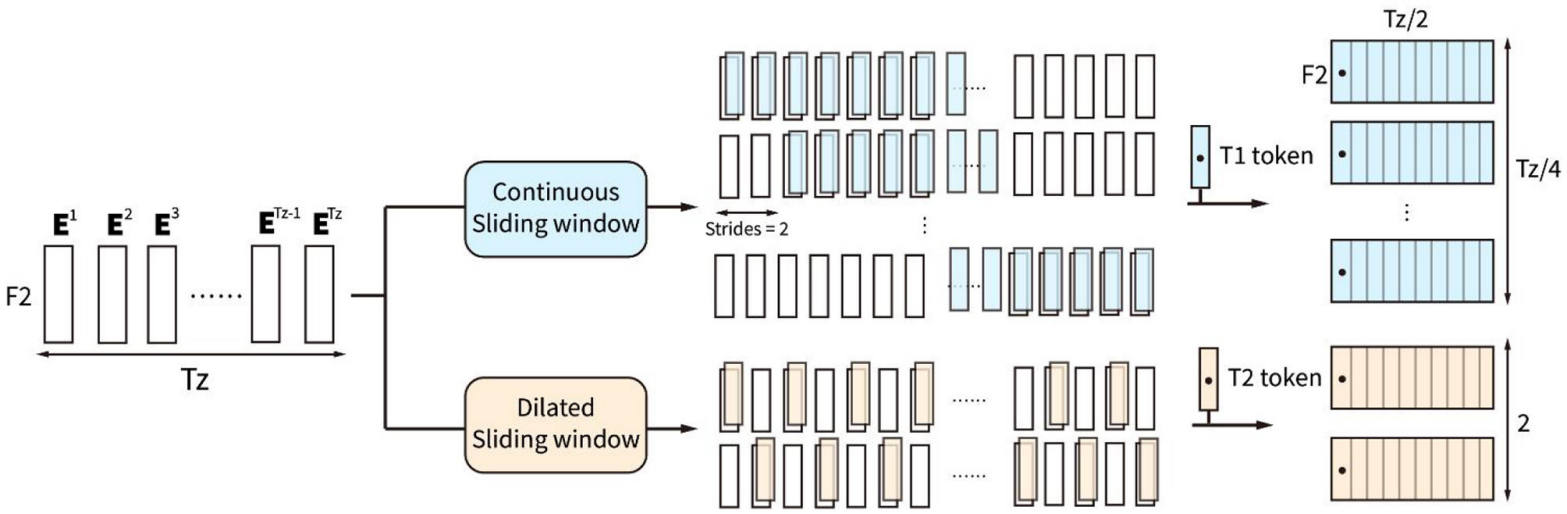
Sliding window block consists of two types of sliding windows, which are continuous sliding window and dilated sliding window.

The sliding window block with two types of sliding windows is proposed to further integrate the high-level spatiotemporal features from the output token embedding sequence of the convolution block, shown in Figure 3. Parallel continuous sliding windows and dilated sliding windows are proposed in the sliding window block to extract different high-level token sequences from the token embedding sequence **z**_**c**_.

The continuous sliding windows are proposed to find some high-level local effective information by extracting continuous token sequence ${\text{z}}_{s1}^{i}$ with continuous type token *T*1. *T*1 token is the trainable type embedding with *F*_2_ features, which is set as the first token of ${\text{z}}_{s1}^{i}$. ${\text{z}}_{s1}^{i}$ is defined by:


(2) $$z_{s1}^{i}=[E_{t1},E^{2i-1},E^{2i},\cdots ,E^{2i+T_{z}/2-2}],\;1\leq i\leq T_{z}/4$$

${\text{z}}_{s1}^{i}$ is the *i*th continuous token sequence with *T*_*z*_/2 tokens. The first token **E**_*t*1_ is the type embedding for continuous sliding windows. There are totally *T*_*z*_/4 identical **E**_*t*1_ locating at the first token of *T*_*z*_/4 continuous token sequences.

The dilated sliding windows are proposed to find effective global integrated information by extracting the discontinuous token sequence ${\text{z}}_{s2}^{i}$, which is calculated by:


(3) $$z_{s2}^{i}=\{\begin{array}{c} \;\left[E_{t2},E^{1},E^{3},\cdots ,E^{T_{z}-1}\right]\;,\;i=1\\ \;\left[E_{t2},E^{2},E^{4},\cdots ,E^{T_{z}}\right]\;,\;i=2 \end{array}$$

${\text{z}}_{s2}^{i}$ is the *i*th discontinuous token sequence with *T*_*z*_/2 tokens. Tokens in the sequence are interval selected. The first token **E**_*t*2_ in the sequence is the trainable type embedding for a dilated sliding window, with *F*_2_ features. Combining the two types of token sequences extracted by the sliding window block, the combined sequence **z**_*s*_ = [**z**_*s*1_, **z**_*s*2_], containing *T*_*z*_/4 continuous and 2 discontinuous token sequences, is the output to the attention block.

### 2.3. Attention block

**Figure 4:**
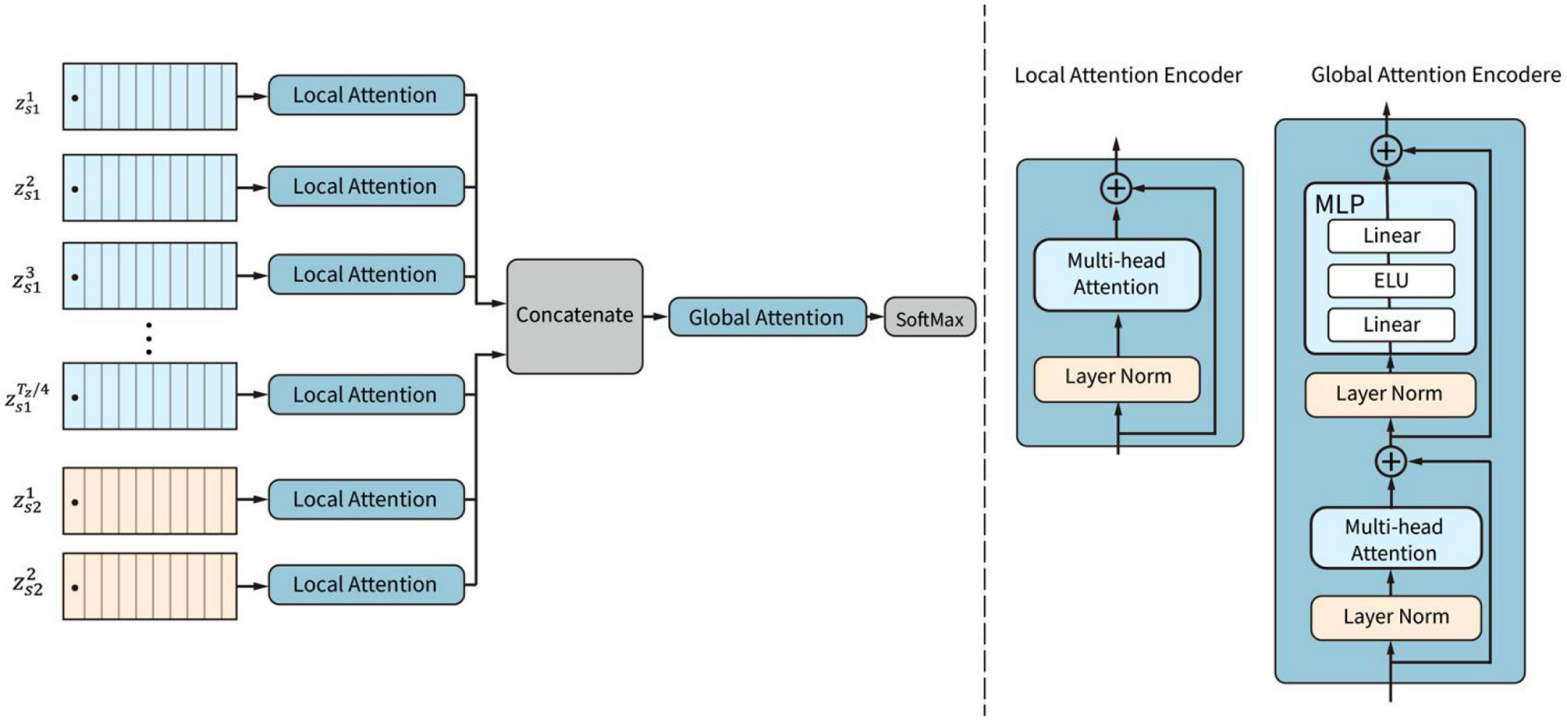
The structure of the attention block. Local attention subnetworks are used to weight the tokens within each sequence. Then global attention is employed to adaptively weight all the tokens in all sequences.

The attention mechanism is a powerful structure for capturing dependencies in images or sequential data, including EEG data. The attention block in the CSANet is proposed with a two-stage attention mechanism, including local attention and global attention, as shown in Figure 4. The local and global attention subnetworks are based on EEG classification. We first classify the data using two types of sliding windows. The continuous sliding window splits the data continuously and in sequence, enhancing the characteristics of continuous data. The dilated sliding window splits the data at intervals, which allows for the extraction of data characteristics over larger spaces and longer time periods. The segmented data is then passed through local attention to extract small-scale local features. After merging these features, they go through global attention to extract global features. Global attention and local attention differ not only in the data they analyze but also structurally. Both use Multi-head Attention, but the difference lies in the additional MLP layer in the global attention module because the data is classified after global attention.

The local attention subnetwork is designed to adaptively weight the tokens in each sequence by capturing local dependencies among the tokens. The global attention subnetwork is designed to adaptively weight all the tokens according to the global attention of all tokens in all sequences.

**Figure 5:**
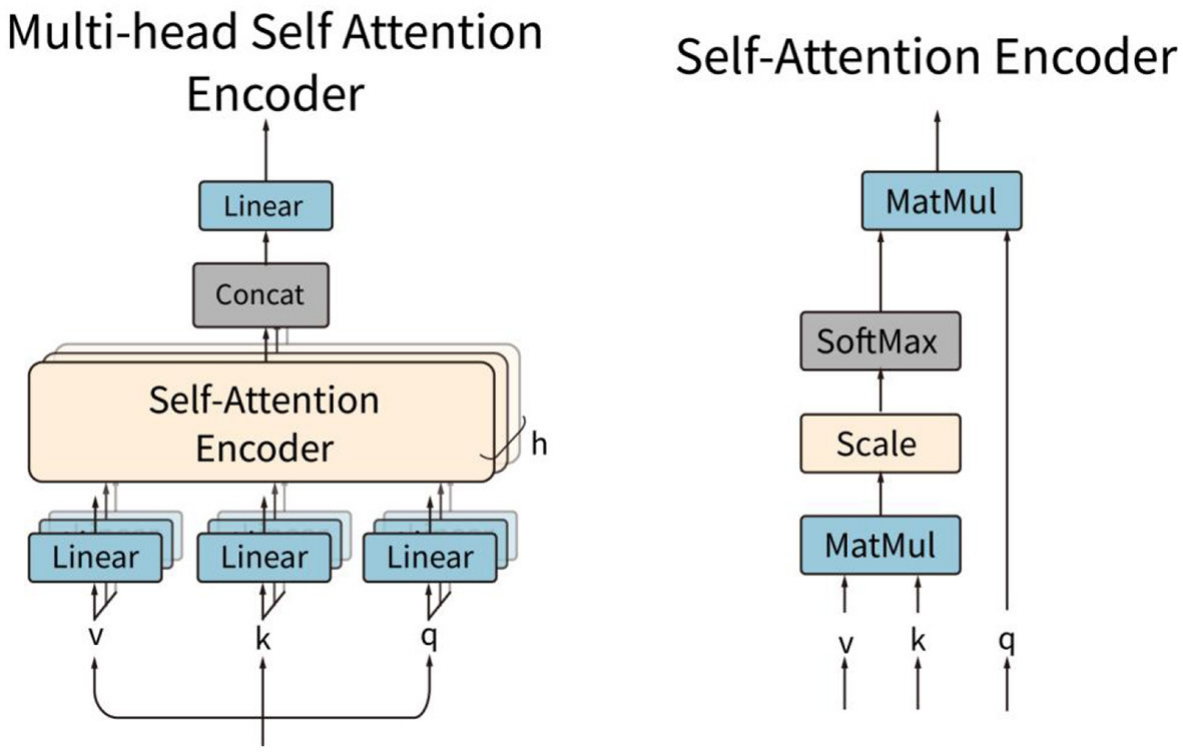
Structures of the multi-head self-attention encoder and the self-attention encoder.

The local attention subnetwork is composed of *L* parallel encoders. Each of the encoders contains a layer-normalization and a multi-head self-attention (MSA) layer, as shown in Figure 5. The local attention subnetwork processes the input token embedding sequence **z**^*i*^ by:


(4) $$z_{l}^{i}=MSA(LN(z^{i})+z^{i},\;z^{i}\in z_{s},\;1\leq i\leq L,\;L=T_{z}/4\;+\;2$$

${\text{z}}_{l}^{i}$ is the weighted token sequence by the local attention subnetwork. *L* is the number of sequences. **z**^*i*^ is a raw sequence extracted in the sliding window block. *LN* is the layer normalization operation. *MSA* is the multi-head self-attention function, which is composed of several self-attention encoders. A single self-attention encoder calculates the correlation weights of all the features in the token embedding. For each self-attention, three trainable matrices **W**_*q*_, **W**_*k*_, ${\text{W}}_{v}\;\in {\mathbb{R}}^{F_{2}\times F_{2}}$ were defined. These matrices transform the input features **z** into **q, k**, and **v** vectors, respectively.


$$q=zW_{q},\;k=zW_{k},\;v=zW_{v}\;$$

For each head *i*_*h*_, the matrices **q, k, v** are further transformed by linear transformations matrices **W**_*q, i*_, **W**_*k, i*_, ${\text{W}}_{v,i}\;{\mathbb{R}}^{F_{2}\times D_{h}}$ to obtain **q**_*i*_*h*__, **k**_*i*_*h*__, **v**_*i*_*h*__, respectively. The dimension of the head is *D*_*h*_.


(5) $$q_{i_{h}}=qW_{q,i},\;k_{i_{h}}=kW_{k,i},\;v_{i_{h}}=vW_{v,i}$$

For each head *i*_*h*_, **q**_*i*_*h*__ and **k**_*i*_*h*__ calculate the scaled dot-product attention by dividing by the square of *D*_*h*_ and then by the SoftMax function. Finally, the output weights in one head are obtained by multiplying with **v**_*i*_*h*__:


(6) $$A(z)=SoftMax\left(\frac{q_{i_{h}}k_{i_{h}}{}^{⊤}}{\sqrt{D_{h}}}\right)v_{i_{h}}$$

**A**(**z**) is the self-attention weight for one head self-attention encoder. The weights of the multi-head self-attentions are composed of each head of self-attention weight. The weight is used to scale the raw token embedding sequence by:


(7) $$\begin{array}{l} MSA\left(\text{z}\right)=\text{z}\cdot \left[A_{1}\left(\text{z}\right);A_{2}\left(\text{z}\right);\cdots \;;A_{h}\left(\text{z}\right)\right] \end{array}$$

*MSA*(**z**) is the weighted token sequence of multi-head self-attention. *A*_*i*_(**z**) is the self-attention weight calculated by the self-attention encoder. *h* is the number of heads.

All the weighted token embedding sequences ${\text{z}}_{l}^{i}$ are then concatenated into one token sequence **z**_*a*_ with *T*_*z*_/2 × (*T*_*z*_/4 + 2) tokens. All the tokens are weighted by the local attention subnetwork and put into the global attention subnetwork.

The global attention subnetwork contains two layers of normalization, namely, a MSA and a fully connected layer. The structure of the multi-head self-attention is identical to the structure of the local attention subnetwork, which weights the tokens by:


(8) $${z^{\prime }}_{a}=MSA(LN(z_{a}))+z_{a}$$

**z**_*a*_ is the global token sequence. ${{\text{z}}^{\prime }}_{a}$ is the output global token sequence weighted by the global attention subnetwork. Finally, a layer normalization layer, a fully connected layer with the ELU function, and a fully connected layer with the SoftMax function are used to calculate the probabilities of different MI categories as follows:


(9) $$y=sofmax(MLP(MLP(LN({z^{\prime }}_{a})))+{z^{\prime }}_{a})$$

*MLP* is the linear projection operation of the fully connected layers. **y** is the output probability of MI categories.

### 2.4. Experimental settings

**Figure 6:**
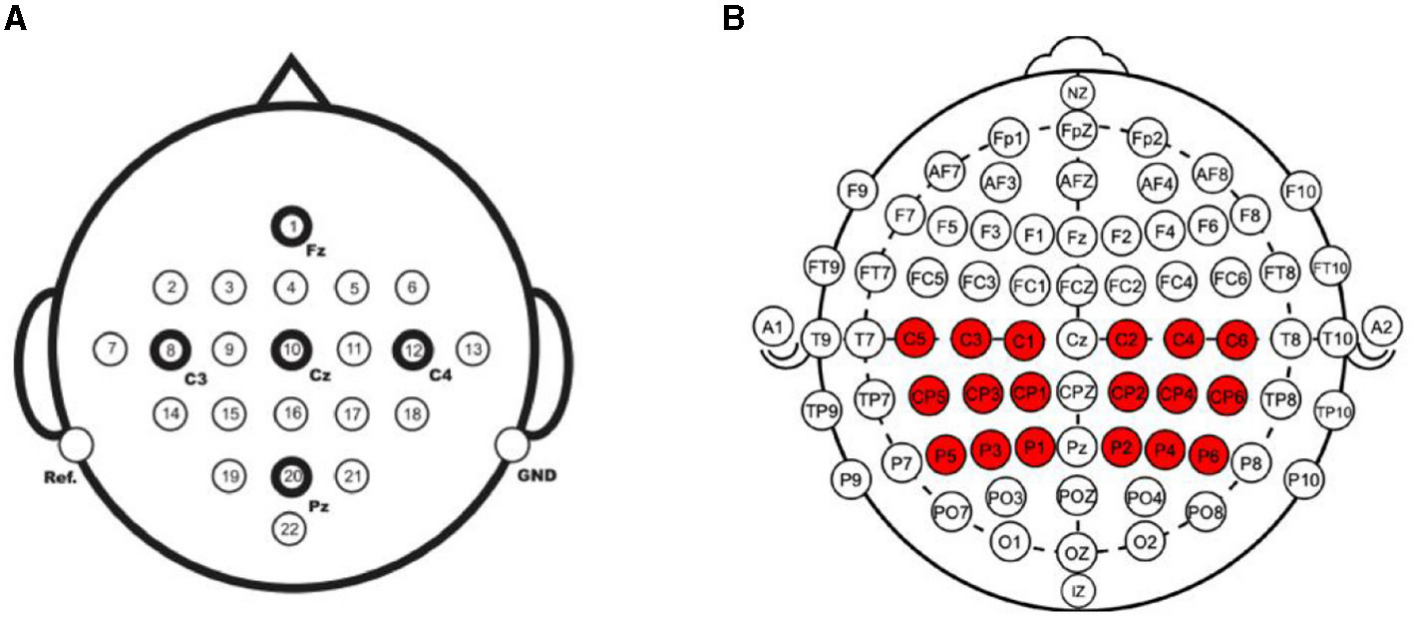
The electrodes used in the two MI-EEG datasets. **(A)** The BCI Competition IV-2a dataset collected EEG signals from 22 electrodes. **(B)** The Physionet MI-EEG dataset collected signals from 64 electrodes. In total 18 of them were used for MI classification.

CSANet was trained and evaluated in the within- and between-individual classification tasks in two public MI-EEG four classification datasets: the BCI Competition IV-2a (BCI-2a) dataset (Brunner et al., 2008) and the Physionet MI-EEG dataset (Goldberger et al., 2000). The details of the two datasets are presented in Table 1, and the electrodes used in the two datasets are depicted in Figure 6.

**Table 1:** Two datasets and the methods within and between individual classification tasks of the two experiments.

		**BCI-2a dataset**	**Physionet dataset**
Created in		2008	2004
Subjects		9	109
Sessions		2 (one for training, one for testing)	1
Trials		288	84
MI task types		Left hand, right hand, foot, and tongue	Left hand, right hand, foot, and tongue
Electrodes		22	18 of 64
Sampling rate		250 Hz	160 Hz
Time of one motion		4.5s	4s
Time points		1,125	640
Within-individual classification task	Training set	First session	90% data
	Test set	Second session	10% data
	Method	-	10-fold cross-validation
Between-individual classification task	Training set	All subject second session	Data of 9 or 10 individual
	Test set	Second session	Data of the other 100 or 99 individuals
	Method	Leave-one-out cross-validation	11-fold cross-validation

The BCI-2a dataset was created by Graz University of Technology in 2008. It consists of recordings from nine healthy subjects who underwent two sessions. The first session was used for training, while the second session was used for testing. Each session contained 288 trials, with each trial comprising one of four motor imagery tasks: movements of the left hand, right hand, foot, and tongue. MI-EEG signals were recorded using 22 Ag/AgCl electrodes (10–20 international standard lead system) and were sampled at 250 Hz with a bandpass filter between 0.5 Hz and 100 Hz (with a 50 Hz notch filter enabled). In the within-individual classification task, the same training and testing data as the original competition were used, with the first session as the training set and the second session as the test set. In the between-individual classification task, the second session of all the subjects was used to be trained and tested. Leave-one-out cross-validation was employed in the classification task. At each validation, the data of one subject was selected as the test dataset, and the data of the other eight individuals were selected as the training dataset. The performance metrics were calculated across all individuals.

The Physionet MI-EEG dataset was recorded using the BCI2000 system according to the international 10-10 system and consists of recordings from 109 individuals. Each individual performed 84 trials comprising four types of MI tasks involving the left fist, right fist, both fists, and feet. There are 21 trails for each type of MI task. Each MI event lasted for 4 s, and the signals were sampled at 160 Hz. Each MI event had 640 time points. In the experiments, we used the electrode methods referenced in two papers (Singh et al., 2019; Xie et al., 2022). For motor imagery, the main location where the brain generates responses is the motor cortex, which is where the 18 electrodes we selected are located. Signals from 18 electrodes near the motor cortex (C1–C6, CP1–CP6, P1–P6) were used in the model training and testing. In the within-individual classification task, 10-fold cross-validation was conducted for the data of each individual. At each validation, 10% of the data was used as the test dataset, and the remaining 90% was set as the training set. The classification accuracy was computed for each test set, and the average test metrics were calculated and reported. In the between-individual classification task, 11-fold cross-validation was conducted to evaluate the performance of the proposed model. In each validation, the data of 9 or 10 individuals were taken as the test set, and the data of the other 100 or 99 individuals were taken as the training set. The average performance metrics were calculated and reported.

Besides the performance experiments of within- and between-individual classification tasks, an ablation experiment was also conducted to test the effects of the proposed type token, sliding window, local attention subnetworks, and global attention subnetwork on the proposed CSANet. We also compared the performance of the proposed model with that of SOTA models in the same within- and between-individual classification tasks. We also extracted the extracted features and utilized the t-distributed Stochastic Neighbor Embedding to evaluate if the extracted features could be distinct in different types of MI tasks.

All the experiments were conducted on a machine with 12 CPU cores, one NVIDIA GeForce RTX 3090, Ubuntu 18.04, Python 3.8, and TensorFlow 2.4. The hyperparameters used in the two datasets are shown in Table 2. Table 3 shows the detailed structures of the proposed models for different datasets and the output of each layer in the BCI dataset and Physionet model. All the models were trained for 1,000 epochs with an Adam optimizer at a learning rate of 0.009 and a batch size of 64. The cross-entropy was used as the loss function in all experiments. These training hyperparameters are determined by manual tuning in the training sets.

**Table 2:** The hyperparameters of three blocks used in the BCI-2a and the Physionet MI-EEG datasets.

**Convolutional block**	
Temporal filters (***F***_**1**_)	16
Depth multiplier (B)	2
Channel conv filters (***F***_**2**_)	32
First pooling size (***P***_**1**_)	8
Second pooling size (***P***_**2**_)	7
Dropout rate	0.3
**Sliding window block**	
Number of windows (***T***_***z***_/**4**+**2**)	7
Dropout rate	0.3
Attention block	
Head size (***D***_***h***_)	8
Dropout rate	0.5

**Table 3:** Detailed description of the proposed model, where C, number of channels; T, number of Time points; **F**_**s**_, sample rate; **F**_**1**_, number of temporal filters; B, number of convolution filters; **P**_**1**_, number of first pooling filter; **P**_**2**_, number of second pooling filter.

**Layer type**	**Maps**	**Kernel size**	**Output**	**BCI output**	**Physionet output**
**Convolutional block**					
Temporal convolutional	*F*_1_	(1, *F*_*s*_//4)	(*F*_1_, T, C)	(16, 1,125, 22)	(16, 640, 18)
Channel convolution	B	(C, 1)	(*B*×*F*_1_, T, 1)	(32, 1,125 1)	(32, 640, 1)
Average pooling		(1, *P*_1_)	(*B*×*F*_1_, T// *P*_1_, 1)	(32, 140, 1)	(32, 80, 1)
Local convolutional	*B*×*F*_1_	(1, 16)	(*B*×*F*_1_, T// *P*_1_)	(32, 140)	(32, 80)
Average pooling		(1, *P*_2_)	(*B*×*F*_1_, T// *P*_1_//*P*_2_)	(32, 20)	(32, 11)
**Sliding window block**			*T*_*z*_ = *T*// *P*_1_/*P*_2_		
Continuous sliding window dilated sliding window	*T*_*z*_/4	(*B*×*F*_1_, *T*_*z*_/2)	(*T*_*z*_//4 + 2, *B*×*F*_1_, *T*_*z*_//2)	(7, 32, 10)	(4 ,32, 5)
Class token			(*T*_*z*_//4 + 2, *B*×*F*_1_, *T*_*z*_/2 + 1)	(7, 32, 11)	(4 ,32, 6)
**Attention block**					
Local attention			(*T*_*z*_/4 + 2, *B*×*F*_1_, *T*_*z*_/2 + 1)	(7, 32, 11)	(4 ,32, 6)
Concatenate			(1, *T*_*z*_/4 + 2, *B*×*F*_1_, *T*_*z*_/2 + 1)	(7, 32, 11)	(4 ,32, 6)
Global attention			(1, *T*_*z*_/4 + 2, *B*×*F*_1_, *T*_*z*_/2 + 1)	(7, 32, 11)	(4 ,32, 6)
**Fully connected layer**					

### 2.5. Performance metrics

The accuracy and Kappa scores were used as the evaluation metrics of the performance in all performances, which were commonly used in the EEG signal classification tasks. The accuracy is calculated by:


(10) $$ACC=\frac{\sum_{i=1}^{N_{c}}TP_{i}}{N}\;(10)$$

*ACC* is the accuracy. *N* is the number of samples in the training or test dataset, and *TP*_*i*_ is the number of true positives (correctly predicted positive samples) in class *i*. *N*_*c*_ is the number of MI task categories. For both datasets, *N*_*c*_ = 4. The range of accuracy is between 0 and 1; higher accuracy means a better model. Kappa is the measurement of consistency between two variables. In the experiments, it was used to measure the consistency between the true class labels and the predicted class labels. It is defined by:


(11) $$\kappa =\frac{1}{N_{c}}\sum_{o=1}^{N_{c}}\frac{P_{o}-P_{e}}{1-P_{e}}$$

κ is the calculated Kappa score. *P*_*o*_is the observed consistency rate for the class *o*, and *P*_*e*_ is the expected consistency rate by chance.

## 3. Experimental and results

### 3.1. Results of the ablation experiment

The ablation experiments were conducted to assess the efficacy of the proposed type token, sliding window, local attention subnetworks, and global attention subnetworks in the CSANet model for within-individual classification tasks on the BCI-2a dataset. The results are presented in Table 4.

**Table 4:** The results of ablation experiments.

**Model no**.	**Sliding window block**		**Attention block**		**Accuracy (%)**	**κ**
	**Sliding window**	**Type token**	**Local attention**	**Global attention**		
1	X	X	X	X	81.25	0.750
2	X	X	X	✓	83.02	0.775
3	✓	X	X	X	81.11	0.748
4	✓	X	✓	X	81.94	0.759
5	✓	✓	X	X	81.54	0.756
6	✓	X	X	✓	83.33	0.778
7	✓	✓	X	✓	82.99	0.773
8	✓	✓	✓	X	82.64	0.768
9	✓	X	✓	✓	83.68	0.782
10	✓	✓	✓	✓	84.08	0.784

The token represents the two types of sliding windows in the sliding window block.

There is no separate ablation experiment for the type token in the sliding window block because the model needs to use the sliding window before adding the classification token. Therefore, the type token needs to be employed simultaneously with the sliding window. The same thing happens with the local attention. In the table, Model 1 represents the most fundamental model that solely employs CNN without a sliding window or self-attention mechanism, indicated by crosses in all columns of the table. Models 2 and 3 exclusively utilize individual modules, namely global attention and sliding window, respectively. Meanwhile, Models 4–9 integrate different modules in diverse ways. Based on the results presented in Table 4, it is evident that the incorporation of a global attention subnet has a significant positive impact on model performance. Specifically, Model 2, which solely utilizes the global attention subnet, demonstrates an accuracy improvement of 1.77% compared to Model 1, which does not employ any proposed methods. The incorporation of both sliding window and global attention mechanisms in Model 6 yields a modest yet significant improvement in accuracy, with an increase of 0.21%. It is worth noting that while the inclusion of type tokens in Model 5 leads to a decrease in accuracy, the integration of the local attention subnet in Model 10 achieves an impressive accuracy rate of 84.08%, surpassing that of Model 6 by 0.75%. The type token method is found to be more effective when used in combination with the local attention subnet, as illustrated in Model 8, which exhibits a 0.7% increase in accuracy as compared to Model 4. The combination of global and local attention proves to be highly effective, resulting in significant performance improvements. For instance, Model 9 shows an increase in accuracy of 1.74% as compared to Model 4. Model 10 has a 2.83% higher accuracy rate than Model 1, indicating that our final proposed model with all modules significantly outperforms the original model. In summary, the results of our ablation experiments demonstrate that the proposed type token, sliding window, local attention subnet, and global attention subnet all have a positive impact on the performance of the MI-EEG classification task.

### 3.2. Results of the public datasets

The proposed CSANet method underwent training and testing for within- and between-individual classification tasks using the BCI-2a and Physionet-MI datasets, respectively. Its performance was subsequently compared with that of other state-of-the-art (SOTA) models.

#### 3.2.1. Results of the BCI-2a dataset

The proposed model was initially evaluated on the BCI-2a dataset through individual experiments, wherein the MI-EEG data of nine participants were separately trained and subsequently validated on a test set. The results obtained are presented in Table 5. In comparing the proposed CSANet model with three other SOTA models, including EEGNet (Lawhern et al., 2018), EEG-TCNet (Ingolfsson et al., 2020), and TCNet Fusion (Musallam et al., 2021). It was evident that the former outperformed the other models with an accuracy improvement ranging from 0.4 to 4.4%, ultimately reaching an overall accuracy of 84.1%. This outcome serves to highlight the proposed model's superior learning and prediction capabilities, particularly for individual motor imagery EEG signal patterns, relative to existing models. The standard deviation (SD) of the accuracy is computed to two decimal places, and the kappa values are in decimal form. In our proposed model, the standard deviation of the accuracy is 9.11, which is slightly lower than the other models, but the difference is not substantial. The kappa value of 0.127 is also not significantly different from the other models.

**Table 5:** CSANet was compared to other models in the within-individual classification task of the BCI-2a dataset across nine subjects.

	**Proposed (CSANet)**		**EEGNet**		**EEG-TCNet**		**TCNet fusion**	
**Individual**	**Acc (%)**	**κ**	**Acc (%) (%)**	**κ**	**Acc (%) (%)**	**κ**	**Acc (%)**	**κ**
1	86.11	0.814	88.57	0.851	84.07	0.796	90.74	0.871
2	70.58	0.608	66.02	0.553	66.32	0.553	70.67	0.603
3	95.13	0.941	95.11	0.943	94.11	0.927	95.23	0.933
4	80.63	0.611	73.61	0.653	72.61	0.638	76.75	0.680
5	84.38	0.791	75.46	0.677	76.06	0.688	82.24	0.767
6	69.79	0.651	64.20	0.529	62.90	0.501	68.83	0.589
7	94.10	0.921	90.36	0.873	89.96	0.871	94.22	0.923
8	89.93	0.865	85.83	0.818	84.76	0.802	88.92	0.858
9	93.75	0.916	86.57	0.821	85.49	0.810	85.98	0.811
Average	84.08	0.784	80.59	0.741	83.34	0.776	83.73	0.780
SD	9.11	0.127	10.48	0.139	10.09	0.137	9.23	0.123

SD, is standard deviation.

**Figure 7:**
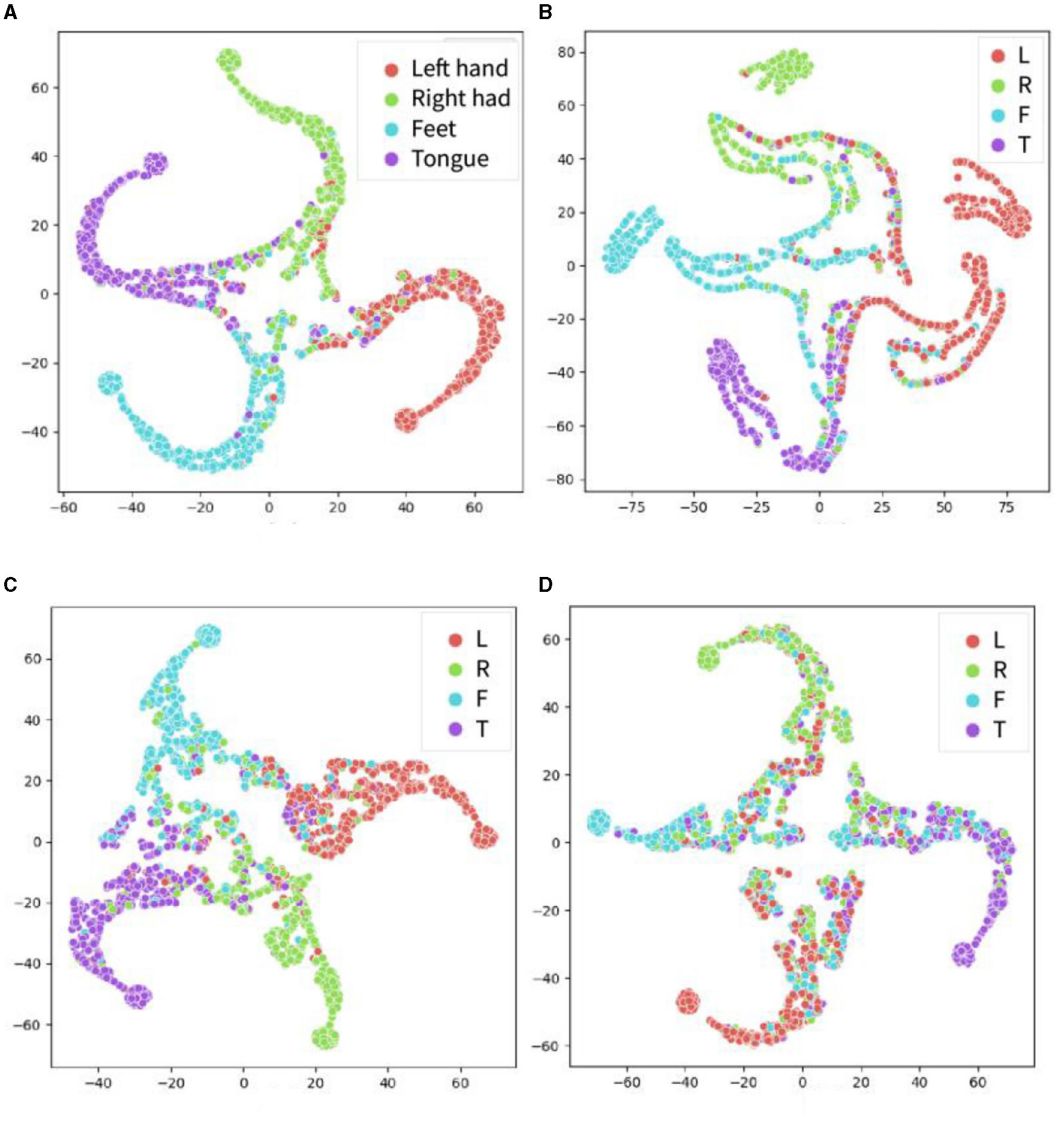
The t-SNE visualization results for the extracted features from the four models for the within-individual classification tasks of BCI-2a dataset. The proposed model CSPNet is shown in **(A)**. **(B)** EEGNet that appears to have less accurate classification performance than the other three models. **(C)** EEG-TCNet. **(D)** TCNet fusion. Four classes include motor imagery of left hand (red), right hand (green), foot (blue), and tongue (purple).

The t-distributed Stochastic Neighbor Embedding (t-SNE) method was used to visualize the extracted features in the proposed models and other models, as shown in Figure 7. In recent years, t-SNE has emerged as a popular tool for data visualization owing to its ability to preserve the local structure of high-dimensional data. Specifically, t-SNE maps high-dimensional data points to a lower-dimensional space while simultaneously preserving the pairwise similarities between them. In this experiment, the features in the global attention layer are extracted and visualized using t-SNE. The horizontal and vertical axes do not possess any physical significance; rather, they represent the two primary components following data dimensionality reduction. During the t-SNE mapping process, these axes are selected to optimize the preservation of local structures within the original high-dimensional dataset (i.e., points that are proximal in high-dimensional space remain so after dimensionality reduction). The principal objective of a t-SNE diagram is data visualization. Based on the visualization results, it is evident that the features extracted by the proposed model exhibit superior distinguishability in the projection of the four categories as compared to other models. Notably, the features extracted by EEGNet demonstrate the poorest distinguishability.

**Figure 8:**
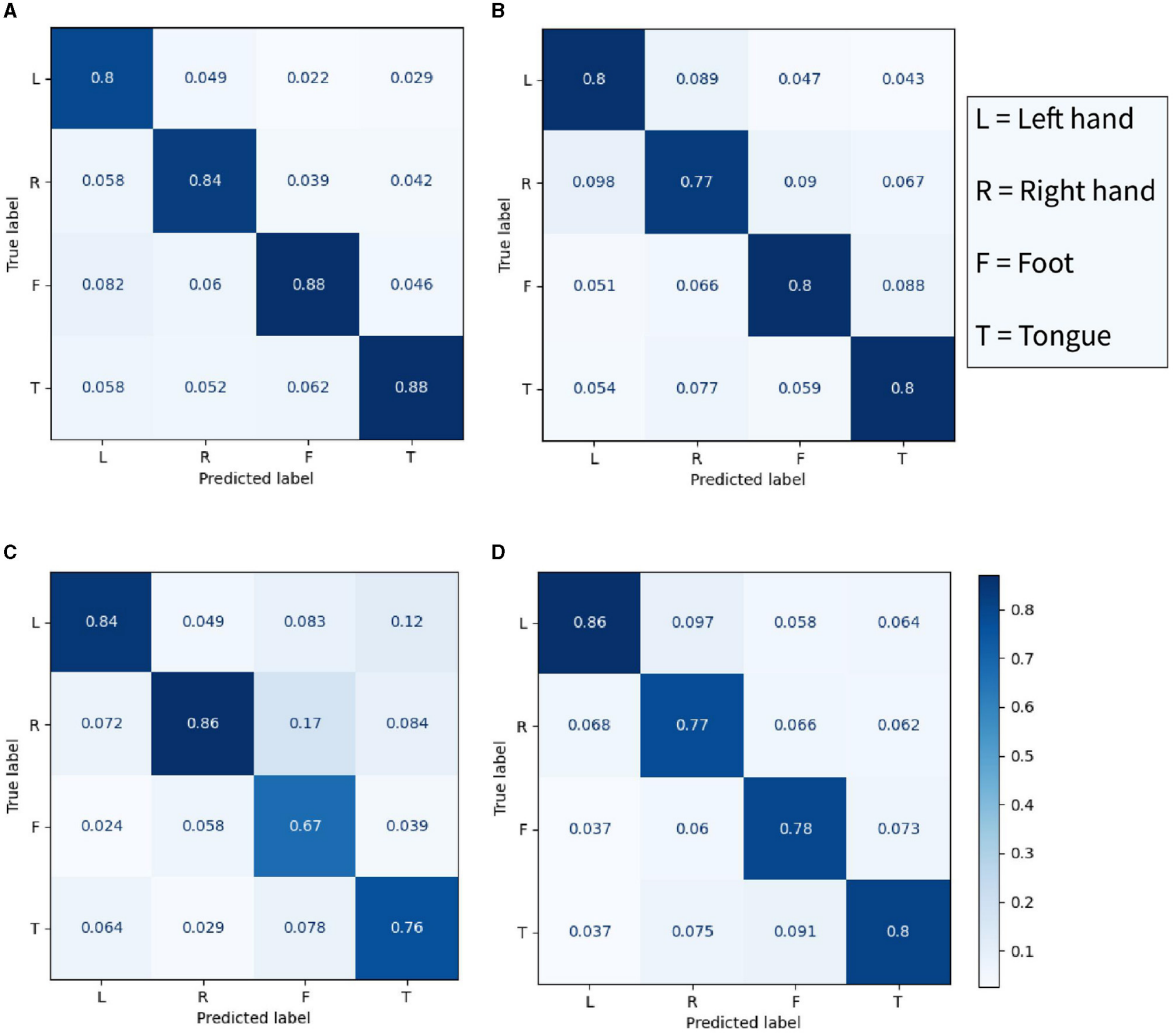
The confusion matrix of four classes in the within-individual classification task of the BCI-2a dataset. **(A)** The proposed CSANet, while **(B–D)** respectively represent EEGNet, EEG-TCNet, and TCNet fusion.

In Figure 8, the accuracy of four models in four categories was compared. The best performance in left-hand motor imagery classification recognition was achieved by TCN Fusion, with an accuracy of 86%. Similarly, an accuracy of 86% was achieved in right-hand classification imagery recognition by EEG-TCNet. The proposed CSANet demonstrated excellent performance in foot and tongue motor imagery classification, with accuracies of 88% for both. This indicates that the accuracy of recognizing the activity of the somatotopic area of the unilateral motor cortex is higher in the proposed model, resulting in a significant improvement in the recognition accuracy of foot and tongue motor imagery. However, the improvement in the classification and recognition accuracy of left- and right-hand motor imagery by the proposed model was not as significant, with accuracies of 80 and 84%, respectively. This result suggests that there may be a need to improve the processing and discrimination of information regarding left-right brain symmetry by the proposed model.

**Figure 9:**
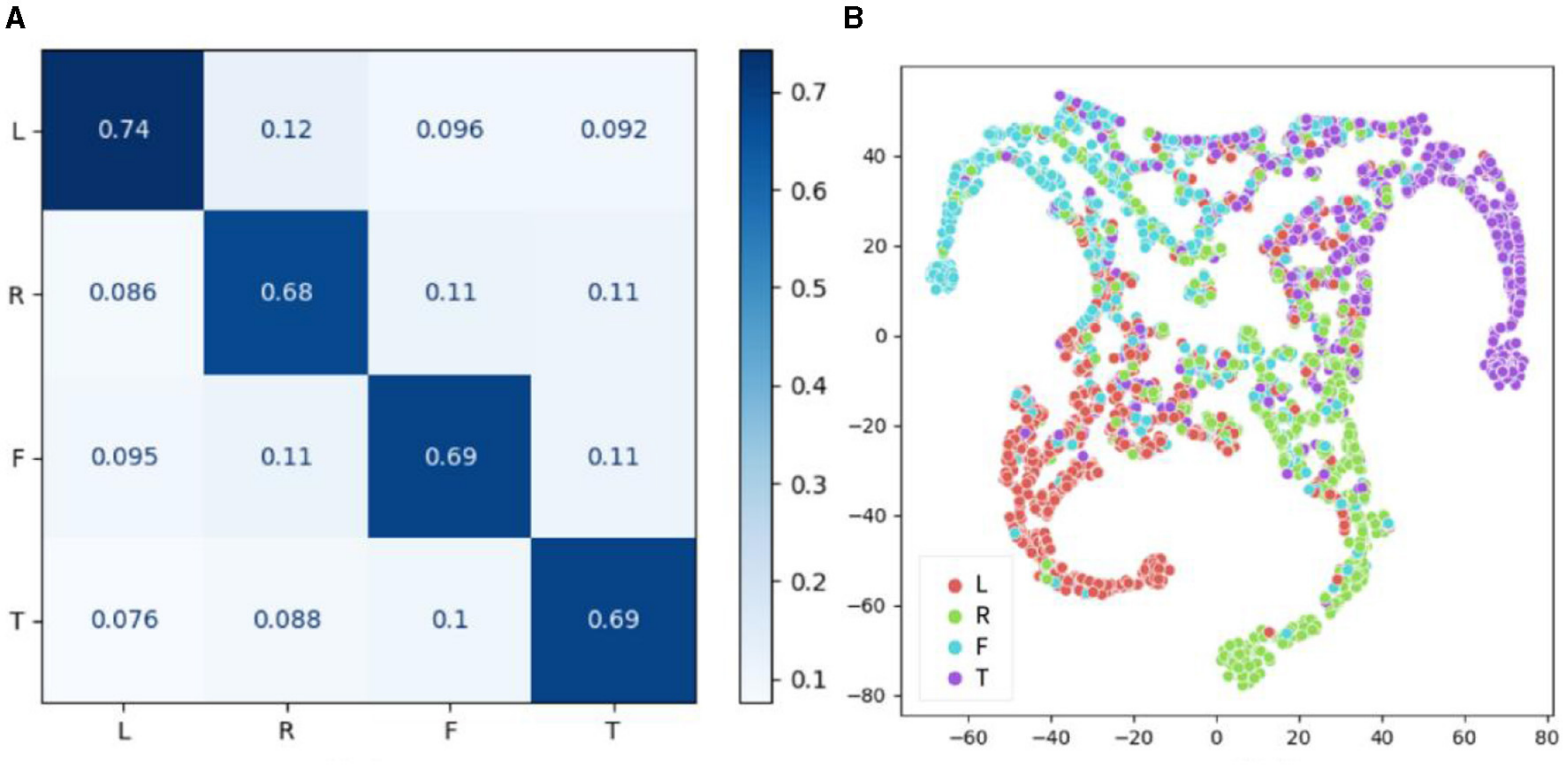
Performance results of CSANet in the between-individual task on the BCI-2a dataset. **(A)** The confusion matrix of four classes. **(B)** The visualized t-SNE results.

In addition to the individual classification task, the between-individual classification performance of the proposed model on the BCI-2a dataset was also evaluated, as shown in Figure 9. The confusion matrix revealed that the accuracy of the four categories was relatively consistent in the inter-individual classification task, with recognition accuracies of 74, 68, 69, and 69% for left hand, right hand, foot, and mouth motor imagery, respectively. Notably, the model demonstrated the highest classification accuracy for left-hand motor imagery, while the classification accuracy for other limb motor imagery was generally similar, resulting in an overall classification accuracy of 70.81%, which was lower than that of the within-individual classification task. These findings suggest that individual specificity still has a certain impact on the model's classification performance. However, the brain signals for left-hand motor imagery were found to be more distinguishable than the other three types of motor imagery signals, and this distinction was found to be cross-individual. The t-SNE results were found to be generally consistent with the confusion matrix results.

CSANet was evaluated against state-of-the-art (SOTA) models on both individual and inter-individual classification tasks using the BCI-2a dataset. The overall mean accuracy and kappa values of the model were compared to those of other models, as presented in Table 6. A total of nine other deep learning models that were also tested on the BCI-2a dataset were compared. The proposed model achieved a higher accuracy of 84.08% and a kappa value of 0.784 in the within-individual classification task compared to other models. An improvement in accuracy of 0.35% and an increase in kappa value of 0.004 were observed. Additionally, the proposed model exhibited the best performance in between-individual classification, with an accuracy of 70.81% and a kappa value of 0.610. Compared with other models, the proposed model improved accuracy and kappa value by 0.23% and 0.002, respectively. Accuracy is mainly affected by the overall performance of the model. And the proposed model is robust. It is noteworthy that the proposed model, which incorporates local and global attention and enriches feature types through multi-type sliding windows, demonstrated improved performance in the MI-EEG signal classification task compared to other attention-based models. This is a testament to the effectiveness of the various sub-modules proposed in CSANet for MI-EEG signal classification.

**Table 6:** Comparing against SOTA models on accuracy (%) and kappa value **κ** on the BCI-2a dataset for both within- and between-individual four-class classification tasks.

**References**	**Method**	**Accuracy (within, %)**	**κ (within)**	**Accuracy (between, %)**	**κ (between)**
Schirrmeister et al. (2017)	CNN	74.31	0.66	-	-
Lawhern et al. (2018)	EEGNet	80.59	0.741	68.79	0.584
Hassanpour et al. (2019)	DBN-AE	71.0	-	-	-
Amin et al. (2019)	Multi-layer-CNN and MLP	75.0	-	55.3	-
Ingolfsson et al. (2020)	EEG-TCNet	83.34	0.776	69.52	0.594
Zhang et al. (2020)	Attention graph cnn	-	-	60.1	-
Musallam et al. (2021)	TCNet_Fusion	83.73	0.780	70.58	0.608
Amin et al. (2022)	Attention-inception CNN and LSTM	82.84	-	-	-
Altuwaijri et al. (2022)	Attention multi-branch CNN	82.87	0.772	69.10	-
This work	CSANet	84.08	0.784	70.81	0.610

#### 3.2.2. Results of the Physionet MI-EEG dataset

In addition to the BCI-2a dataset, a comparison was conducted between the proposed CSANet model and other SOTA models on the Physionet MI-EEG dataset, covering both within-subject and between-subject tasks. The results presented in Table 7 demonstrate that the highest accuracy in both intra-subject and inter-subject classification tasks was achieved by the proposed model, with accuracies of 92.36 and 70.56%, respectively. Notably, an improvement ranging from 4.22 to 24.16% and from 2.02 to 11.98% was observed compared to other SOTA models.

**Table 7:** Comparing results on the Physionet MI-EEG dataset for both within- and between-individual four-class classification tasks.

**References**	**Method**	**Within accuracy**	**Between accuracy**
Ma et al. (2018)	RNN	68.20	-
Pinheiro et al. (2018)	RNA	74.69	-
Dose et al. (2018)	CNN	80.38	58.58
Karácsony et al. (2019)	CNN	76.37	-
Wang et al. (2020)	EEGNet	-	68.20
Ali et al. (2022)	ConTraNet CNN-Transformer	-	65.44
Hou et al. (2022)	GCN	88.14	-
Xie et al. (2022)	Transformer	-	68.54
This work	CSANet	92.36	70.56

**Figure 10:**
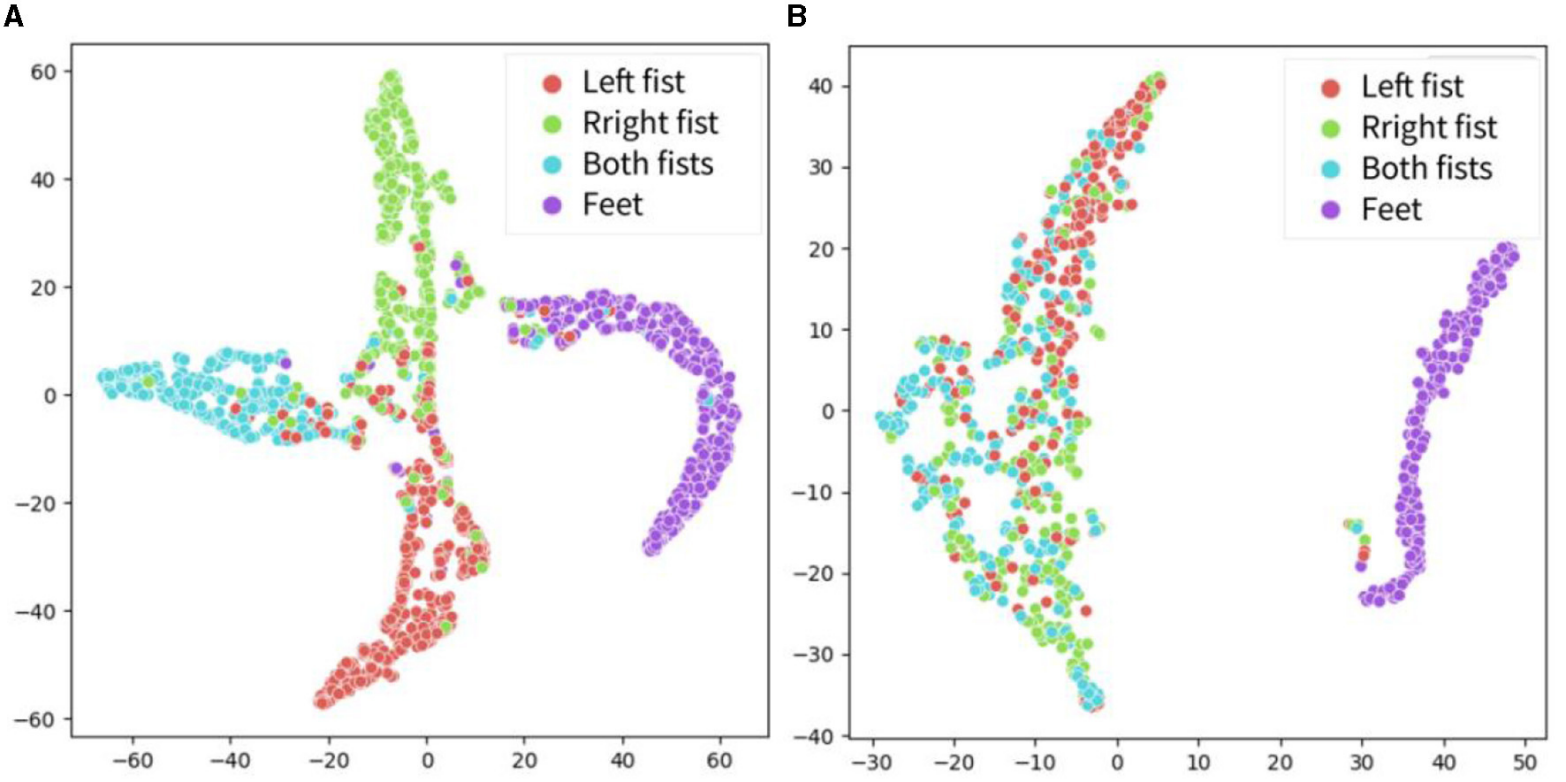
The t-SNE visualization for the four-dimensional features on the Physionet MI-EEG dataset. Include left fist, right fist, both fist, and feet. **(A)** Features in the within-individual classification task. **(B)** Features in the between-individual classification task.

The features of the proposed CSANet model calculated on the test set in within- and between-individual tasks were extracted, and the t-SNE method was utilized to display the feature projection of four types of motor imagery in the dataset: left fist, right fist, both fists, and feet. As shown in Figure 10, in the within-individual classification task, most of the samples exhibited high feature distinctiveness, indicating that the effective features of the four types of motor imagery could be accurately distinguished by the proposed model without considering the specificity of individual EEG signals. However, in the between-individual classification task, the feature overlap of motor imagery of the left fist, right fist, and both fists was high and the distinctiveness was low, thereby impeding accurate classification. Notably, the feature distinctiveness of motor imagery of feet was higher compared to the other three types of samples. These findings suggest that the proposed model exhibits higher recognition accuracy for activity in the somatotopic area of the unilateral motor cortex but still lacks processing of symmetric neural activity information for hand movements in the bilateral brain areas.

## 4. Discussion

In this study, we proposed a CSANet model that integrates multi-scale convolutional feature extraction, a multi-perspective sliding window, and a two-stage attention mechanism to address the challenges in classifying motor imagery EEG signals.

Our ablation experiments on each sub-module of the proposed method revealed that the introduction of the global attention module significantly improved the classification performance of the model on MI-EEG data. Moreover, the methods of global and local feature extraction based on sliding windows and local multi-head attention showed significant impacts on the model's classification performance. Although the introduction of the type token method may have certain side effects on the model in the case of single activation, its combination with local multi-head attention significantly improved the model's performance. This enhancement may be attributed to the fact that the role of type token is influenced by other dimensional features in different data environments, which static deep learning models without local attention mechanisms cannot handle effectively. Consequently, by integrating a local multi-head self-attention mechanism and endowing the model with the ability to learn dynamic weights of type token, the model's performance can be greatly improved.

In the study, the proposed CSANet model was compared with nine other deep learning models using the BCI-2a dataset. The results showed that a higher accuracy of 84.08% and a kappa value of 0.784 were achieved by the model in the within-individual classification tasks, surpassing the performance of other models. Furthermore, the best performance in between-individual classification was exhibited by the model, with an accuracy of 70.81% and a kappa value of 0.610. On the Physionet MI-EEG dataset, the highest accuracy was achieved by the model compared to other state-of-the-art models in both within- and between-individual classification tasks, with accuracies of 92.36 and 70.56%, respectively. These accuracies represented significant improvements of 4.22 and 2.02%, respectively. The classification of MI-EEG signals remains a challenging topic in current research, and limited improvements have been shown in previous studies on algorithms for MI-EEG signal classification. For instance, EEGNet-TCNet was proposed by Ingolfsson et al. (2020) on the BCI-2a dataset, achieving an accuracy of 83.34%, which represented a 2.74% improvement over previous models. Subsequently, Musallam et al. (2021) proposed TCNet_Fusion, which achieved an accuracy of 83.73%, a 0.39% improvement. Altuwaijri et al. (2022) proposed a CNN combined with an attention model, achieving an accuracy of 82.87%, which was a 0.86% improvement over previous models. On the Physionet dataset, Xie et al. (2022) achieved an accuracy of 68.54%, which was 2.81% higher than the previous CNN model's accuracy of 65.73%. The proposed CSANet model outperforms these studies on both datasets, with improvements of up to 4.22%. Although the improvement is not very significant, the same model has effective results on both datasets, demonstrating the robustness of the CSANet model.

In recent years, self-attention mechanisms have been widely adopted in EEG classification research. For example, Xie et al. (2022) utilized attention mechanisms in both temporal and spatial domains, while many other models integrated CNNs with attention mechanisms for data classification (Altuwaijri et al., 2022). Ali et al. (2022) also employed a combination of CNNs but incorporated the Vision Transformer (Dosovitskiy et al., 2021) in the attention mechanism to introduce position embeddings for feature classification. In addition to CNNs, Amin et al. (2022) achieved remarkable performance by integrating LSTM. Our model utilizes two types of sliding windows to extract features with both continuous and global dimensions. Local and global attention allow for a two-stage dynamic assignment of feature weights, which facilitates the selection of more relevant features. When combined with type tokens, it can extract features more accurately and enhance the robustness of the model, enabling the extraction of important features from different datasets to accurately classify EEG signals. The model's enhancement of classification performance in MI-EEG tasks has been demonstrated in the experiments on two public EEG datasets and has surpassed other methods. Although this improvement is not significant enough, it is at the same level as other work relative to SOTA methods. Through ablation experiments, we have proven the effectiveness of each module. Importantly, our model reduces the individual specificity of EEG signals by recognizing common patterns among subjects. This approach effectively highlights local features while also enabling the application of global features during classification. In this article, four experiments on two datasets used similar hyperparameters to achieve good performance, which also demonstrates the robustness of our proposed method and extracted features.

The features extracted by the proposed model were visualized using the t-SNE method, and the confusion matrix was calculated, as shown in Figures 8–10. It was observed that for the within-individual classification task in BCI-2a, the classification results for foot and tongue motor imagery were significantly improved by our proposed model compared to other models. In the between-individual classification task, a higher accuracy in classifying left-hand motor imagery was achieved by the model than in other limb parts, but the overall classification result was lower. In the Physionet MI-EEG dataset, a very high feature discriminability was observed for the individual classification task. The discriminability of features for left fist, right fist, and both fists in between-individual MI classification was not high, but foot motor imagination was effectively distinguished. These results suggest that the classification effect of the proposed model in the between-individual task was lower than that in the within-individual task due to the influence of individual specificity. Furthermore, it was noted that the t-SNE analysis of the individual classification in BCI-2a and the inter-individual classification in Physionet MI-EEG demonstrated that the model had a better recognition effect on the body mapping EEG signals of the unilateral brain motor area, but the discriminability of the activation of bilateral brain information still needs to be improved. The overall improvement may be attributed to the ability of the proposed model to mine more effective spatiotemporal features and dynamically combine and weigh the features with a two-stage local and global attention mechanism to improve the overall classification performance of MI-EEG signals.

The model proposed in this study can be applied not only to the development of brain-computer interface control systems based on motor imagery but also to the neurorehabilitation evaluation of diseases such as Parkinson's and stroke based on motor imagery. In the evaluation process, MI-EEG signals from healthy individuals can first be trained based on CSANet. Then, the trained model can be used to classify and visually evaluate the motor imagery signals of patients with Parkinson's or stroke who are in the rehabilitation period. If the patient's motor system is severely damaged, the classification accuracy of the MI-EEG model might be lower than that of healthy individuals. Through visual evaluation, the specific accuracy of identification of the patient's limb movement imagination can be determined, and targeted training can be conducted for the parts with lower identification accuracy to quickly improve the patient's recovery effect. When the overall MI-EEG signal classification accuracy of the patient is high, it is indicated that the patient's motor imagery EEG signal pattern is close to that of healthy individuals. It could be estimated that the patient's nervous system has recovered to a certain level of limb movement control according to the conclusions of the mirror neuron system and the theory of embodied cognition.

## 5. Conclusion

The convolutional sliding window-attention network (CSANet) model proposed in the article is composed of novel spatiotemporal convolution, sliding window, and two-stage self-attention blocks. The adaptive feature learning and selection ability of multi-scale information correlations in EEG signals is improved by the type token, sliding window, and local and global multi-head self-attention mechanisms proposed in the model, thereby enhancing the model's classification performance, as demonstrated by the results of the ablation experiment analysis. The model has been demonstrated to outperform existing state-of-the-art (SOTA) models in within- and between-individual classification tasks in two commonly used MI-EEG datasets, BCI-2a and Physionet MI-EEG, with classification accuracies improved by 4.22 and 2.02%, respectively. Based on t-SNE visualization of the model features and confusion matrix analysis, it can be inferred that the proposed model exhibits superior performance in identifying EEG signals in the unilateral somatotopic area, although the discernibility of bilateral brain information activity remains a challenge. Furthermore, this study proposed a plausible neurorehabilitation assessment framework based on the model for mental diseases such as Parkinson's disease and stroke based on motor imagery. In future work, the model would be further improved based on its shortcomings, and experiments would be conducted on MI-EEG data of specific disease patients to demonstrate the neurorehabilitation assessment framework based on the CSANet model.

## Data availability statement

The original contributions presented in the study are included in the article/supplementary material, further inquiries can be directed to the corresponding authors.

## Author contributions

YH designed the model, experiments, and wrote the paper. JZ, BX, XL, and YL participated in the experimental design process with JZ providing expertise in experimental design methods. BX processed the public datasets used in two experiments. XL provided experimental design advice and implementation. YL assisted in interpreting and reviewing the experimental results. ZW supervised and reviewed the research, editing, and revising the paper. HF provided model design ideas and suggestions. SC provided clinical experience and insights in the experiments. All authors contributed to the article and approved the submitted version.
